# Organic electronic transmembrane device for hosting and monitoring 3D cell cultures

**DOI:** 10.1126/sciadv.abo4761

**Published:** 2022-09-16

**Authors:** Charalampos Pitsalidis, Douglas van Niekerk, Chrysanthi-Maria Moysidou, Alexander J. Boys, Aimee Withers, Romane Vallet, Róisín M. Owens

**Affiliations:** ^1^ Department of Physics and Healthcare Engineering Innovation Center (HEIC), Khalifa University of Science and Technology, P. O. Box 127788, Abu Dhabi, UAE.; ^2^ Department of Chemical Engineering and Biotechnology, University of Cambridge, Philippa Fawcett Drive, Cambridge CB3 0AS, UK.

## Abstract

3D cell models have made strides in the past decades in response to failures of 2D cultures to translate targets during the drug discovery process. Here, we report on a novel multiwell plate bioelectronic platform, namely, the e-transmembrane, capable of supporting and monitoring complex 3D cell architectures. Scaffolds made of PEDOT:PSS [poly(3,4-ethylenedioxythiophene):polystyrene sulfonate] are microengineered to function as separating membranes for compartmentalized cell cultures, as well as electronic components for real-time in situ recordings of cell growth and function. Owing to the high surface area–to–volume ratio, the e-transmembrane allows generation of deep, stratified tissues within the porous bulk and cell polarization at the apico-basal domains. Impedance spectroscopy measurements carried out throughout the tissue growth identified signatures from different cellular systems and allowed extraction of critical functional parameters. This platform has the potential to become a universal tool for biologists for the next generation of high-throughput drug screening assays.

## Teaser

An organic bioelectronic platform hosts and monitors 3D cell systems for more predictive and high-throughput biological assays.

## INTRODUCTION

Current understanding of the growth, function, and homeostasis of cells and tissues mainly arises from two-dimensional (2D) cell-based assays using cell monolayers cultured on flat, rigid substrates. While such assays have been applied for both fundamental research and toxicology screening, they do not recapitulate the complex 3D microenvironment found in vivo (*1*). This is reflected in the lack of topographical features and the limited degrees of freedom for spatial organization, resulting in poor cell-cell and cell-ECM (extracellular matrix) interactions (*2*). The inadequacy of 2D cell cultures to generate the required information for drug efficacy and toxicity has been a major contributing factor in high failure rates in the drug discovery process (*3*). Animal studies, on the other hand, despite being regarded as the “gold standard” for preclinical trials, pose several limitations, including physiological differences between species, high cost, lengthy time frames, and ethical concerns (*4*, *5*). To overcome these constraints, efforts have shifted toward improved in vitro systems, termed 3D cell culture models, which can better mimic the physiological architecture of in vivo systems, exhibiting phenotypic and functional characteristics similar to their living tissue counterparts (*6*, *7*). Advances in 3D cell biology have particularly favored the development of these biological models. Spheroids and organoids, for example, represent highly versatile and more physiologically relevant 3D cell models, thanks to their intrinsic properties of better emulating various aspects of the morphology and function of their tissue of origin, both under homeostatic and pathophysiological conditions (*8*, *9*). However, drug development/screening studies with such systems suffer because access to the luminal/apical side is challenging (possible only via microinjection). In addition, these biological systems grow on hydrogel supports, sometimes of not fully defined composition (e.g., Matrigel), which may incorporate the necessary biochemical cues, but not the microtopography and architectural complexity found in the native ECM (*10*).

Introduction of the essential 3D structural features within such confined systems by means of ECM components and 3D spatial cell organization remains challenging. The need for models that allow cell organization in 3D requires the use of tissue-engineered constructs (i.e., scaffolds, hydrogels, and fibrous meshes). These constructs can provide an architecture and environment that more accurately emulates key aspects of human physiology (*11*). Most scaffolds used for 3D cell cultures are based on natural (e.g., collagen and chitosan) or synthetic [e.g., poly(vinyl alcohol) and poly(lactic acid)] polymers. Although these scaffolds capture the 3D architecture, evaluation of living tissues can be challenging because of difficulties in imaging through 3D structures. These limitations can be overcome by using other means of characterization, for example, electrical measurements, provided that the scaffolds are electrically active (*12*). As we have shown recently, conducting polymer (CP) scaffolds can be used as hosting structures for 3D cell cultures and active elements in electronic transducers for label-free monitoring and real-time live-cell sensing (*13*–*19*). As cells grow on the 3D CP scaffolds, they alter the electrical properties of the electrode, thus allowing for dynamic monitoring of biological events related to tissue growth and status (e.g., cell attachment and tissue differentiation).

A widely used cell culture setup for high-throughput in vitro applications (e.g., cell migration, toxicology and drug/nutrient transport) is the Transwell insert. Typically, cells are grown on these filter membranes suspended in culture media. This format also facilitates compartmentalization of the culture (through separation of apical and basal compartments), essential for developing epithelial and endothelial tissues in vitro (*20*, *21*), as well as various types of cocultures (e.g., cancer–immune cell interaction and host-microbe interactions) (*22*). Real-time evaluation of cell/tissue health and integrity within these culture setups has been challenging, with most assays being of end point nature or relying on labeling of biomarkers and analysis of supernatants. Impedance-based cell monitoring has offered a highly valuable alternative approach, particularly for noninvasive and continuous characterization of epi/endothelial barrier integrity by means of transepi/endothelial electrical resistance (TEER) (*23*, *24*). The most common techniques for measuring TEER involve either single-frequency measurements using “chopstick electrodes” (e.g., EVOM) (*23*, *25*) or using electrochemical impedance spectroscopy (EIS), which offers more detailed characterization of cellular health and status, measuring current in a broad frequency spectrum [e.g., cellZscope, nanoAnalytics GmbH (*26*) and various electrodes (*25*, *27*)]. While easy to use, convenient, and, in most cases, standardized and high-throughput, these methods still rely on rigid, high-impedance electrodes (e.g., gold and silicon oxide) placed on either side of the tissues and thus fail to provide the necessary intimate cell-electrode coupling for high-content, sensitive, and accurate cell sensing. Recent attempts have focused on increasing complexity by growing epithelial cells in collagen gels on a multiwell plate with incorporated electrodes for impedance (TEER measurement). However, unlike in the Transwell format, access to the basal compartment is somewhat challenging (*28*).

Here, we demonstrate a bioelectronic transmembrane (e-transmembrane) device that combines a number of desirable properties: (i) the higher throughput nature of the well plate configuration, (ii) Transwell-like operation that allows apical and basal sampling, (iii) the ability to host biologically complex and physiologically relevant 3D cell cocultures, and (iv) real-time monitoring of the models. PEDOT:PSS [poly(3,4-ethylenedioxythiophene):polystyrene sulfonate]–based scaffolds were engineered to function as separator membranes for compartmentalized cell cultures, simultaneously acting as electronic elements for recording cell growth and tissue integrity. Adopting an electrode configuration, the e-transmembrane device can electrically monitor changes associated with the presence of cells, both in the bulk of the scaffold and at the outer interfaces. To validate the compatibility and functionality of our system, the e-transmembranes were cultured in situ with human tissue-specific cells and combinations, modeling both the intestinal epithelium and the vascular endothelium. Impedance spectroscopy measurements, carried out throughout the culturing period on the e-transmembranes, allowed us to distinguish various cell impedance profiles to assess cell growth and to extract barrier function parameters (i.e., TEER). In addition, we demonstrate the organic electrochemical transistor (OECT) mode of operation, highlighting the potential of the transmembrane device to operate in multiple device formats and extract different electrical readouts. Given its compatibility with well plate formats (i.e., high-throughput assays), as well as its 3D biomimicking capabilities, our e-transmembrane platform could serve as a highly useful tool for drug discovery.

## RESULTS

### Fabrication and electrical characterization of e-transmembrane platform

**Fig. 1.**
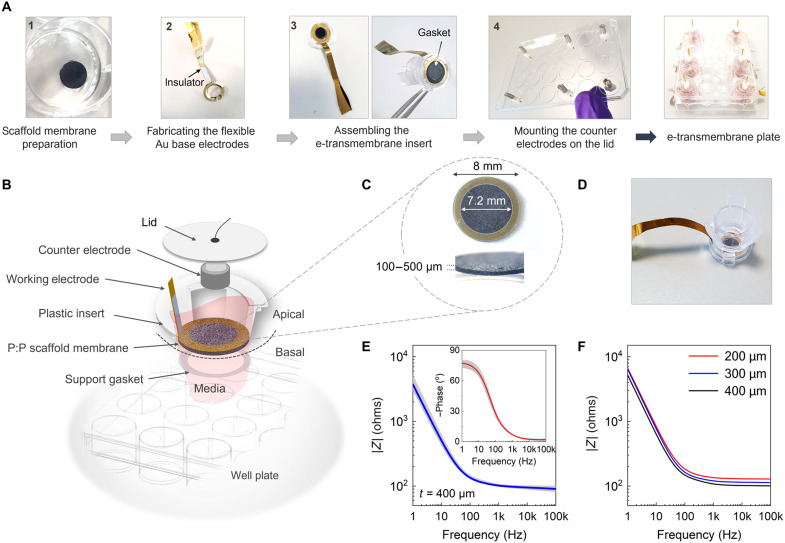
The concept of the e-transmembrane platform. (**A**) Photographs showing the processing steps for the fabrication of the e-transmembrane platform. (**B**) Schematic illustration of the bioelectronic insert showing the various critical components of the device. The sectional structure clearly shows the basal and the apical domains and the geometrical features of the electrodes. The working electrode (WE) module is composed of a gold (Au) base electrode attached to the PEDOT:PSS scaffold membrane. The counter electrode (CE) is a high surface area metal woven mesh attached to the lid of the well plate. The insert setup shown here is compatible with a 24-well plate configuration. (**C**) Optical micrograph of a PEDOT:PSS e-transmembrane. The thickness of the membrane typically ranges between 100 and 500 μm, and its diameter is 8 mm. The aperture is defined by the inner diameter of the Au O-ring electrode and, in the present configuration, is approximately 7.2 mm. (**D**) Photograph of the fully assembled device. (**E**) Electrochemical impedance spectroscopy measurements showing the Bode magnitude (|*Z*| − *f*) and the corresponding phase plots (insert) measured from multiple devices (of 400 μm thickness, two batches, *N* = 5). The solid blue and red lines represent the generated averaging curves. (**F**) Bode plot spectra of e-transmembrane electrodes of various thicknesses (200, 300, and 400 μm). Note that EIS measurements were carried out in cell culture medium.

To fabricate the e-transmembrane device, three key modules are engineered sequentially and assembled for adaptation in a well plate configuration (Fig. 1A): (i) CP scaffold membrane, (ii) electrical interconnects for capturing electrochemical readouts, and (iii) plastic insert components. The first step involves lyophilization of the PEDOT:PSS solution in a 48-well plate and the subsequent sectioning of the resulting scaffolds using a vibrating blade microtome. The quality and the consistency of the scaffold slices are determined by adjusting the speed and the oscillation amplitude of the blade during the sectioning step (see Materials and Methods). Commercially available inserts are then engineered to host the scaffold membrane that lies between the basal and the apical compartment of the well. Next, flexible gold O-ring electrodes are patterned and attached on the plastic insert to ensure direct contact with the PEDOT:PSS scaffold membranes. An insulating layer of polydimethylsiloxane (PDMS; or parylene C) is deposited over the gold surface (step 2 of Fig. 1A), covering the part that is in direct contact with the electrolyte to avoid possible interference in electrochemical measurements. For the assembly of the Transwell insert, plastic O-ring gaskets of fixed aperture (~7.2 mm) are placed and concentrically “sandwich” the scaffold membrane (step 3; Fig. 1A). Last, the platform is completed by integrating high-surface platinum (Pt; or stainless steel) mesh counter-electrodes and wiring on the well plate lid (step 4; Fig. 1A), allowing for in situ electrical monitoring, without immersing external circuitry that may disturb the geometry of the electrochemical cell or compromising sterility. A detailed schematic of the platform and corresponding images of the PEDOT:PSS-based membranes and the fabricated insert are shown in Fig. 1 (B to D).

### Electrical device operation

The e-transmembrane system is a two-terminal electrochemical device that can be operated in electrode configuration for electrochemical impedance spectroscopy (EIS) measurements. As shown in the Bode magnitude (*|Z| − f*) plots in Fig. 1E, the device exhibits an ohmic behavior at the high- to mid-frequency region (100 to 0.5 kHz), as indicated by the line parallel to the *x* axis, and a capacitance-dominated regime in the low-frequency range (<100 Hz) (*29*). This is also identified in the phase plot (see inset), where a transition from ~0° (resistive) to nearly 80^o^ (predominantly capacitive) can be observed. As expected, the PEDOT:PSS-membrane electrodes exhibit low impedance magnitude, with values typically ranging between 100 and 135 ohms at 1 kHz (for 400-μm-thick slices measured in cell culture medium). In addition, an overall decrease in the impedance magnitude is observed with increasing the thickness/volume of the membranes, as shown in Fig. 1F. This can be attributed to the decrease in the electrolyte resistance and the double-layer impedance, as they are both inversely proportional to the surface area of the electrode. In the absence of any other interface atop of the PEDOT:PSS scaffold membranes, our electrodes can be adequately described by a |*R*_s_ − *C*_el_| or |*R*_s_ − CPE| (constant phase element) circuit, with *R*_s_ being the spreading resistance at the electrode/electrolyte interface and *C*_el_ being the associated capacitance. The CPE expresses the deviation from an ideal capacitor and, in 3D electrodes, can be attributed to the surface roughness, porosity, variability in thickness, and nonuniform charge distribution (*30*). Note that because of the large surface-to-volume ratio, the PEDOT:PSS scaffold membranes may pose slight variations in the impedance magnitude between sets of samples. However, these variations can be limited by precisely controlling the properties of the starting solution and the processing parameters of the scaffold membranes. Note also that in the presence of cell culture or physiological medium, electrodes are exposed to a complex electrolyte mixture containing growth factors, salts, glucose, and amino acids, among other components, which may result in alterations in their electrochemical behavior. As shown in the comparative EIS plots [phosphate-buffered saline (PBS) versus cell culture medium] in fig. S1A, our membrane electrodes when measured in PBS, exhibit a shift toward lower frequencies accompanied with a slight drop in the overall impedance magnitude. To exclude potential misinterpretation of the measured signal in the presence of cell culture medium, we have also investigated the temporal performance stability, as shown in fig. S1B. It was found that the devices exhibit negligible variations in the impedance magnitude (ΔΖ < 20 ohms at 1 kHz) after 2 weeks in cell culture medium, with no substantial alterations in their spectra profile.

Going one step further with the device engineering, we have also explored the use of PEDOT:PSS scaffold membranes in an organic electrochemical transistor (OECT) configuration, as shown in fig. S2A. This unique 3D OECT essentially follows a vertical architecture with the PEDOT:PSS scaffold membrane being “sandwiched” between two gold O-ring electrodes that serve as source and drain terminals (fig. S2B). The transistor characteristics are shown in fig. S2 (C and D). The transistor device follows a typical depletion mode of operation, with distinct transition from linear to saturation regime, as can be seen in the output curves. The source-drain current is modulated by the doping-dedoping process upon control of the voltage at the gate electrode, similar to 2D planar OECTs (*31*). Given the large volume of the PEDOT:PSS, our OECT device exhibits high level of source-drain current and transconductance values of approximately 72 mS (fig. S2E). Although preliminary, one can also envision a bimodal operation of this platform using both transistor and electrode configuration, thus enriching the amount of data extracted from the biological system. While the electrode mode represents the current state of the art to study cellular barrier properties at the critical basal and apical biointerfaces (*23*, *25*), the vertical transistor architecture may be useful for sensing changes in the intraporous bulk space of the membranes. Here, we focus exclusively on the electrode mode of operation for benchmarking our device with cellular systems. Nevertheless, we intend to investigate in depth the transistor mode of operation in future studies, focusing on performance optimization and various measuring schemes.

### Morphological properties of e-transmembranes

**Fig. 2.**
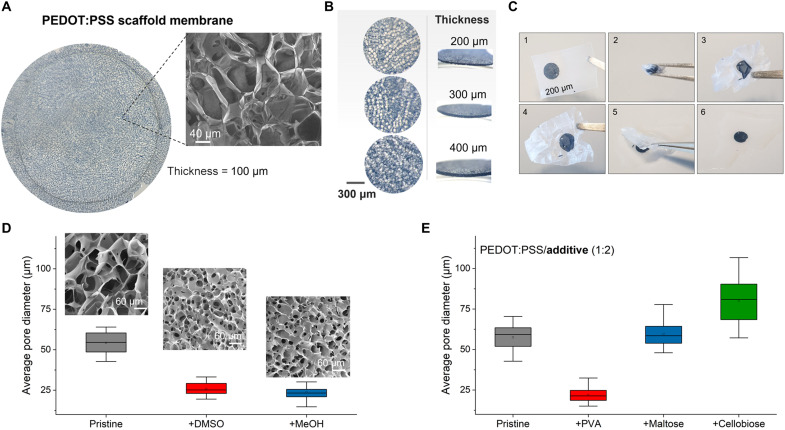
Morphological properties of the PEDOT:PSS e-transmembranes. (**A**) Optical micrograph of a 100-μm-thick scaffold membrane and SEM image of the corresponding pore morphology. (**B**) Optical micrographs showing top and side views of PEDOT:PSS e-transmembranes of various thicknesses (200, 300, and 400 μm). (**C**) Sequential photos showing the macroscopic effects of crumpling-unfolding test on PEDOT:PSS e-transmembranes. To perform the test, a 200-μm-thick membrane is attached to a parafilm tape and then subjected to a random macroscopic deformation. (**D**) Tailoring the pore size of the PEDOT:PSS e-transmembranes using inhibitors of the freezing process. Pristine PEDOT:PSS solution is mixed with dimethyl sulfoxide (DMSO) or methanol (MeOH) at 1 and 2.0% (v/v) concentration, respectively. The graph shows the corresponding mean pore diameter approximated from at least *N* = 20 pores using image analysis in the SEM images. (**E**) e-transmembrane composites based on mixtures of PEDOT:PSS with polyvinyl alcohol (PVA) or with oligosaccharides (maltose and cellobiose). The ratio of PEDOT:PSS/additive is 1:2. The graph shows the mean pore diameter approximated from at least *N* = 20 pores.

While vibratome sectioning is capable of generating micrometer-thin slices, in the case of PEDOT:PSS scaffolds, we are more limited because of the presence of the pores, meaning that the thinnest size achieved without compromising the structural integrity of the slices is approximately 100 μm. Figure 2A shows an optical micrograph and scanning electron microscopy (SEM) measurement of such a slice. The image reveals a honeycomb-like macroporous morphology consisting of a network of interconnected open pores, with some pores spanning the substrate in the *z* direction. We note that as the thickness of the membranes increases (>200 μm), more pore “lattices” are stacked, resulting in a loss of pore orientation. The PEDOT:PSS e-transmembranes exhibit an average pore size (diameter) of 57.5 (±7.4) μm and a porosity of approximately 78%, consistent with micro–computed tomography measurements performed in scaffolds of the same composition (*17*). For use in tissue engineering models, these values fall within the characteristic range for sufficient cell infiltration and growth (*32*). Typically, the thickness of the e-transmembranes ranges between 100 and 500 μm (see Fig. 2B), allowing sufficient scaffold hydration and transport of nutrients and oxygen, even under static culture conditions. While scaffold slices as thin as 100 to 200 μm are semitranslucent, there are limitations associated with the cell seeding yield and their handling during cell culture maintenance. For our multiculture cell experiments, the 400- and 500-μm-thick e-transmembranes were found to be the most suitable, as they provide adequate space for ECM-secreting cells (i.e., fibroblasts) to fill the intraporous space and generate a favourable support for seeding other tissue types (i.e., epithelium and vascular).

Mechanical properties of 3D constructs strongly influence cell-substrate interactions and, as a consequence, the associated cell morphology and functions (*33*). Typically, materials with Young’s modulus values within the range of 0.1 to 50 kPa are sufficient for cell cultures (*34*). Previous work from our group and others have shown that PEDOT:PSS scaffolds of the same formulation exhibit a Young’s modulus as low as 37 kPa in wet state and 68 to 90 kPa in dry state (*18*, *35*), thus rendering them suitable for cell culture experiments. On the other hand, handling ultrathin and soft, porous structures can be challenging, and even mild external stresses may affect their structural integrity. As a proof of concept of the durability of the e-transmembranes, we perform a crumpling test, where a 200-μm scaffold slice is folded multiple times and unfolded back, as shown in Fig. 2C. Unexpectedly, no significant macroscopic defects are observed, while the e-transmembrane is seen to completely recover its original shape after immersing it in water.

Previous reports have demonstrated that CP scaffold properties, such as pore size, morphology, and density, can be tailored on demand by changing the lyophilization parameters and/or the composition of the solution before processing (*13*, *16*, *18*, *35*, *36*). In the following section, we explore different modification routes for tailoring the morphological properties of PEDOT:PSS e-transmembranes. Although, for tissue engineering purposes, the pore size achieved with PEDOT:PSS may be adequate/optimal, future use of the scaffolds, (e.g., for monitoring of growth of bacterial cells or algae) may benefit from the ability to tailor the pore size.

### Tailoring pore size and morphology

A common practice to modify the pore size of freeze-dried scaffolds is to change the freezing temperature, with higher freezing points typically resulting in smaller average pore diameter (*37*). Motivated by the work of Jiang *et al.* (*38*), we explored an alternative modification route that involves the use of freeze inhibitors, such as dimethyl sulfoxide (DMSO) or methanol (MeOH), mixed with the pristine PEDOT:PSS solution. As shown in Fig. 2D, this leads to a decrease in the mean pore diameter, from 57.5 ± 7.4 μm (pristine sample) to 25.8 ± 4.1 μm (1% DMSO, v/v) and 23.7 ± 4.7 μm (2.0% MeOH, v/v), respectively, although no significant changes in the pore shape and structure are observed. Notably, these values were extracted via image analysis of SEM images, thus representing rough estimates of the actual pore diameter values.

We further investigated the development of composite e-transmembranes based on PEDOT:PSS/polymer [such as polyvinyl alcohol (PVA)] and PEDOT:PSS/oligosaccharides (such as maltose and cellobiose). PEDOT:PSS/PVA is a commonly used system for bioelectronic interfaces and sensors (*39*, *40*), while PEDOT:PSS/saccharides complexes have recently shown very promising properties for use in 3D cell cultures, as they combine conductivity and biocompatibility (*16*). For that purpose, before the freeze-drying process, pristine PEDOT:PSS solution is mixed with PVA (40 mg/ml in H_2_O) or with maltose (20 mg/ml in H_2_O) or cellobiose (40 mg/ml in H_2_O) in a 1:2 (v/v) ratio. Figure 2E shows the estimated pore size of the resulting e-transmembrane composites. In the case of PEDOT:PSS/PVA, a significant decrease in the mean pore diameter is measured (21.4 μm), most likely because of the increase in the viscosity that, in turn, may hinder the ice crystal formation during the freeze-drying process. In addition, PEDOT:PSS/PVA e-transmembranes exhibit different geometry with pores characterized by sharp edges and corners (fig. S3A). On the other hand, the PEDOT:PSS/oligosaccharides show pore morphology similar to the pristine sample (fig. S3, B and C) despite the apparent increase in the mean pore diameter. Last, the biocompatibility of the composite e-transmembranes was assessed by culturing telomerase immortalized fibroblasts (TIF), labeled with red fluorescent protein (RFP) for 5 days. As shown in the SEM images in fig. S3 (D to F), fibroblasts appear to follow the topography of the porous surface with elongated actin filaments bridging the pores of the scaffolds. PEDOT:PSS/PVA show a dense accumulation of fibroblasts over the top surface; however, the presence of small pores and rough edges seems to hinder their elongation, as can be observed in the immunofluorescence image in fig. S3G. Both PEDOT:PSS/oligosaccharides demonstrate fibroblasts with elongated body spreading on top and within the pores, as confirmed by the image in fig. S3 (H and I). In summary, these experiments show the versatility of our conducting scaffold system for a variety of future applications where modulation of pore size, while remaining biocompatible, is important.

### Modeling e-transmembrane electrode operation

**Fig. 3.**
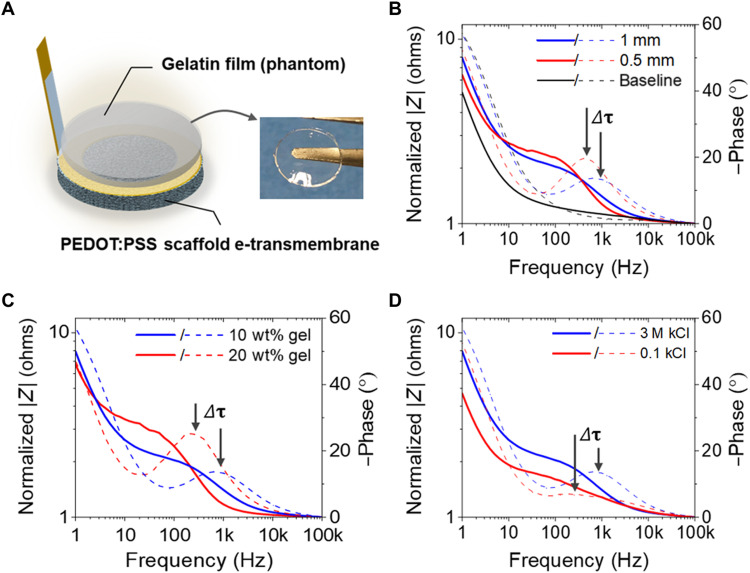
Validating the electrochemical operation of the e-transmembrane device using gelatin phantom inserts. (**A**) Schematic representation of the e-transmembrane with the phantom installed (i.e., a gelatin film clamped onto the apical side of the PEDOT:PSS scaffold). Varying physical and chemical properties of the phantom changes its resistivity or permittivity (capacitance), which will cause a shift in the time constant (τ = *RC*) of the parallel *R*-*C* impedance of the phantom. Comparative impedance Bode plots of the gelatin phantoms (**B**) for various thicknesses (0.5 mm versus 1 mm), showing a time constant to shift to lower frequencies that indicates an increase in the capacitance with decreased thickness. (**C**) Increasing the gelatin content [from 10 to 20 weight % (wt%)] of the phantom electrode increases the permittivity, which increases the capacitance and causes the time constant to shift to a lower frequency. (**D**) Decreasing the KCl concentration from 3 to 0.1 M increases the resistivity, which causes the time constant to shift to a lower frequency. [Solid lines correspond to impedance magnitude (|*Z*|), and dashed lines correspond to impedance phase; black data series correspond to the blank device without the gelatin phantom.]

Before testing the feasibility of the 3D e-transmembrane platform for assessing cell barrier integrity, we validated its operation using a “phantom” layer, where a phantom is an input presented to a system with a known output. In the case of barrier-forming tissue, the expected output is qualitative; hence, while a phantom cannot be used to quantify sensor accuracy, it can assess whether the output of our platform is as expected. Here, an appropriate phantom is any material that impedes ionic flux via an established mechanism, yielding a known impedance, when placed atop or within the PEDOT:PSS scaffold. Gelatin hydrogels, commonly used as phantoms in the development of electrophysiological sensors (*41*) and with well-characterized dielectric and (ionic) conductive behavior, were selected as phantoms here (schematically illustrated in Fig. 3A). Their impedance consists of a resistor in parallel to a capacitor (*42*, *43*), similar to the equivalent circuit model of the impedance of a cellular barrier model to ionic flux. Furthermore, the capacitance and resistance of the gelatin can be varied, where the expected change in impedance is known qualitatively, with *RC* equal to the time constant τ. Thus, increases in either *R* or *C* result in increases in τ, while the features of the impedance curves (e.g., frequency of the knee; start of the magnitude roll-off) shift to lower frequencies. To better highlight the relative change in time constant and the impedance morphology of the phantom, the total measured impedance magnitude of the device is normalized to the high frequency value and the units are converted to decibels (dB), yielding a convenient magnitude range between 1 and 10. This allows a qualitative observation of the morphology/shape of the measured impedance and its relative changes (and the changes in τ) between test cases (thus, the exact value of the impedance magnitude is somewhat irrelevant). The impedance of the device without phantom shows a low-frequency roll-off [fall in magnitude due to the impedance of the capacitive counter electrode (CE)] and a flat mid- and high-frequency interval (Fig. 3B). As expected, when the gelatin film phantom is inserted, the mid-frequency interval exhibits magnitude and phase features characteristic of a parallel *R*-*C* equivalent circuit element (Fig. 3B), similar morphologically to what is seen with barrier model experiments. In this context, barrier tissues such as the epithelium in the gastrointestinal tract, or the endothelial lining of blood vessels, are those with a primary function that is to mediate the passage of molecules from one compartment to another, and barrier models refer to their in vitro counterparts (*29*). In addition, the phantom thickness, gelatin content (Fig. 3C), and electrolyte concentration (Fig. 3D) were varied (relative to a base case) in such a way that either the capacitance or resistance would increase. The results show that the critical frequency (at which we switch from a purely resistive regime to a mixed *RC* regime, indicated by the magnitude knee and associated with the phase peak) of the phantom shifted to lower frequencies, indicative of an increased time constant (*23*), as expected. Together, these findings suggest that the e-transmembrane sensor measures the impedance of the material (or cellular model), integrated with the scaffold, to ionic flux.

### Device validation using different 3D cell culture systems

**Fig. 4.**
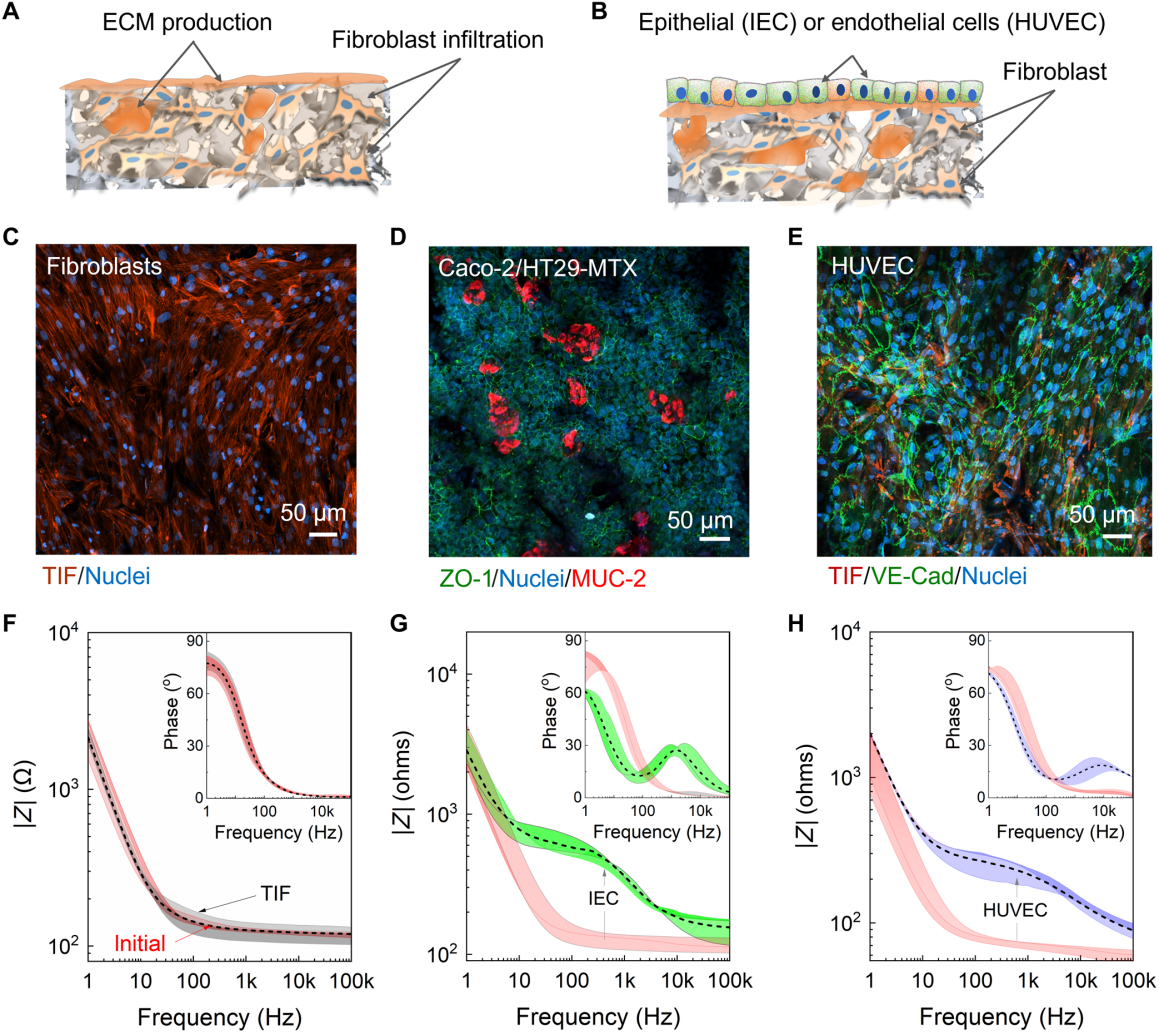
3D cell culture experiments and EIS monitoring using e-transmembrane platform. (**A**) Illustration of tissue formation within the e-transmembrane to support (**B**) 3D epithelial or endothelial models. (**C**) Confocal images of fibroblasts (TIFs; actin cytoskeleton in red), counterstained for nuclei (blue), demonstrating cell infiltration and fibrillar network (21 days of TIF monoculture). (**D**) Immunofluorescence of intestinal epithelium, illustrating ZO-1 protein (green), revealing the formation of the tight junction network and secretion of MUC2 (red), and nuclei (blue; see also fig. S4). (**E**) Immunofluorescence image of human umbilical vein endothelial cells (HUVECs; 14 days) over a fibroblast-cultivated e-transmembrane. Images shows adherens junctions [vascular endothelial (VE)–cadherin in green]. Cells were counterstained for nuclei (blue) visible over a layer of fibroblasts (actin in red; a 500-μm e-transmembrane was used for the HUVEC experiments). Bode plots of the models showing the impedance magnitude from (*N* = 3) different devices and/or experiments. The color bands represent superimposed EIS plots for various devices. The dashed line corresponds to the average curving. (**F**) Fibroblasts (gray band, TIF, 5 days) − initial (uncultured e-transmembranes) and (**G**) IECs (green band, Caco-2/HT29-MTX, 21 days) overlaying fibroblast-cultured e-transmembrane (red band, TIF, 5 days). (**H**) Endothelial cells (blue band, HUVEC, 14 days) overlaying a fibroblast-cultured e-transmembrane (red band, TIF, 5 days). Insets show corresponding phase plot for models for the different devices.

After validating the sensor output, the feasibility of the proposed bioelectronic e-transmembrane platform and its standing with respect to Transwell-based assays are evaluated by developing both an epithelial and an endothelial cell model. The typical e-transmembrane model comprises human fibroblasts grown in the bulk of the scaffold (see illustration in Fig. 4A), with a confluent human epi/endothelial cell monolayer subsequently seeded on top (Fig. 4B). We recently proposed a strategy for engineering complex, stratified tissues in 3D electroactive scaffolds, in which the first step involves cultivation of electroactive substrates with human fibroblasts, serving as a guide for tissue organization upon subsequent integration of other cell types, according to the type of tissue model under development. In that study, we showed that the preseeding of fibroblasts prevents the epithelial cells from invading into the scaffold pores and from creating discontinuous layers of cells lining the pores. We also showed that the fibroblasts secrete copious amounts of ECM proteins that provide a more favorable environment for cell attachment and adhesion; thus, the fibroblasts served as an anchorage point for the subsequently seeded epithelial cells to form the desired barrier tissue in a stratified fashion (*17*). Here, we adopt this strategy to form a baseline for the 3D e-transmembrane models to test whether this 3D cell culture platform can support concurrent development/maintenance and real-time monitoring of human epithelial and endothelial barriers.

For the 3D epithelial model, we use a coculture of intestinal epithelial cells (IECs), comprising Caco-2 cells and HT29-MTX cells (IECs) in a 3:1 ratio. These cells are seeded on the apical surface of the scaffold membranes (on day 5 of the culture), precultivated with fibroblasts, following the same ex situ process; the e-transmembranes of each platform are transferred to dedicated wells of a nontreated 12-well plate, and the cell suspension (either TIFs or IECs) is added on the apical surface of each scaffold. Following a 2-hour incubation period, the scaffold membranes are then transferred back to the e-transmembrane platform (treated/cell culture grade 24-well plate) and complete growth medium is added in both apical and basal compartments. The resulting 3D tricultures are maintained for another 3 weeks (see Materials and Methods and the Supplementary Materials for more details). Note, here, that high-resolution live-cell imaging (e.g., bright-field/phase-contrast imaging) is rather challenging, as the thickness of the scaffolds and the current configuration of the device (electrodes on the well plate lid and suspension of devices in wells) blocks access to the cell culture within the scaffold. Here, to produce high-resolution images of the complex cell cultures, we performed confocal microscopy at the end of the experiment. Specifically, immunofluorescence imaging reveals a continuous monolayer of polarized intestinal cells, anchored on top of the preestablished extensive TIF-derived fibrillar network, intercalating with the porous network of the electroactive substrate (Fig. 4C), as expected. The effective formation of a stratified tissue, with fibroblasts in the bulk and a differentiated monolayer of polarized IECs is shown in fig. S4. Labeling for typical intestinal biomarkers reveals a differentiated intestinal layer with tissue exhibiting typical apical brush border characteristics, including expression of tight junctions [i.e., zonula occludens-1 (ZO-1)] and mucin proteins [i.e., mucin 2 (MUC2); Fig. 4D].

Similarly, human umbilical vein endothelial cells (HUVECs) are used for the development of the 3D endothelial vascular model in the e-transmembranes. As before, e-transmembranes are initially seeded with fibroblasts to provide a supporting layer for generating the vascular membrane. After 5 days, HUVECs are added onto the e-transmembrane devices and cultured under dynamic fluid conditions (orbital shaker) to promote the development of vascular junctions, as fluid shear has been hypothesized to improve vascular phenotypes. We chose to use the terminology dynamic culture rather than perfusion culture, as the stress that is applied is primarily a surface shear stress, given that the scaffolds are affixed within the culture system. However, as the scaffolds are porous, some perfusion-like aspects apply to this culture system as well. By carefully controlling the application of this dynamic approach, we were able to tissue-engineer large-scale endothelial structures that spanned the entirety of the scaffold. The resultant cellular structuring showed good coverage of endothelial cells across the entire surface of the scaffolds with consistent adherent junctions, as demonstrated by the vascular endothelial (VE)–cadherin staining (Fig. 4E). Furthermore, the VE layer was observed to follow the macroscale topography of the e-transmembrane, as confirmed through multilevel imaging on a confocal microscope (fig. S5).

The electrical properties of the different 3D cell cultures in the e-transmembrane platform are assessed using EIS. As shown in the Bode plot in Fig. 4F, the incorporation of fibroblasts results in slight fluctuations around the initial (no cells) curve, without significantly altering the overall impedance spectra profile. However, we have noticed a tendency to decrease the overall impedance magnitude during the first week of cell culture, in line with previous observations (*17*). The magnitude of such change typically ranges between 20 and 50 ohms in the resistive regime (>500 Hz), while in the capacitive-dominated regime (<10 Hz), this range can be hundreds of ohms. One explanation is that fibroblasts as adherent cells stick to the surface of the pores and restructure the electrode by covering the intraporous space and bridging the interporous distance with elongated fibrillar structures. This restructuring may cause a change in the electrode geometry and thus influence its electrochemical operation. Note that this trend tends to saturate after the first days of fibroblasts culture while no effects have been observed in the cell barrier–associated regime. This behavior can be seen in fig. S6, showing the EIS curve evolution during 25 days of TIF-cultured e-transmembranes. A similar trend can be observed in measurements performed using a Transwell filter membrane with commercial impedance system (cellZscope, nanoAnalytics; fig. S7).

The presence of a confluent epithelial layer (after 25 days in total − 21 days of IEC culture) results in an increase in the magnitude of the complex impedance in the mid-high frequency range, corresponding to a polarized, fully differentiated epithelial barrier, lined with mucin (as cross-validated optically; Fig. 4G). Similarly, the Bode phase plot indicates the formation of a time constant in the sub–1-kHz regime, which corresponds to the paracellular resistance. The observed impedance profile is in agreement with literature when using cell monolayers atop planar CP electrodes (*44*). To extract cell-specific parameters from the EIS spectra, the measured impedance data were fitted according to the geometry and the equivalent circuit in fig. S8. Specifically, it is composed of a serial combination of a resistor (*R*_s_, electrolyte resistance), a resistor and a capacitor (or combined CPE for nonideal capacitor) in parallel (*R*_m_||*C*_m_) representing the cell layer, and a combined CPE representing the sum of the capacitance of the working electrode [CPE_W_: Au + (fibroblast-cultured) PEDOT:PSS scaffold] and the capacitance of the CE (CPE_CE_). Fitting the *R* and *C* parameters using the Nyquist plots (as shown in fig. S8A) generates values for the semicircle domain associated with the presence of cell layer. Specifically, the mean value of the net cell resistance is found to be 527.6 ± 116.5 ohms, which corresponds to a TEER value of 215.8 ± 47.6 ohm·cm^2^, while the normalized-to-area cell layer capacitance is 1.47 ± 0.63 μF/cm^2^.

Similar to the epithelial model, the confluent endothelial layer on the e-transmembrane resulted in analogous changes in the impedance spectra as shown in Fig. 4H, albeit slightly less pronounced, given the lower intrinsic paracellular resistance values that have been recorded for HUVECs versus epithelial cells (*25*, *45*). The net cell resistance and capacitance values of the HUVEC model are found to be 244.3 ± 60.6 ohms and 0.30 ± 0.15 μF, respectively, corresponding to a TEER value of 99.9 ± 24.8 ohm·cm^2^ and a normalized-to-area cell layer capacitance of 0.73 ± 0.36 μF/cm^2^. These results were generated from (*N* = 3) different devices and/or experiments. The measured fitting parameters are summarized in table S1.

### Development of a 3D human intestine on plate model

**Fig. 5.**
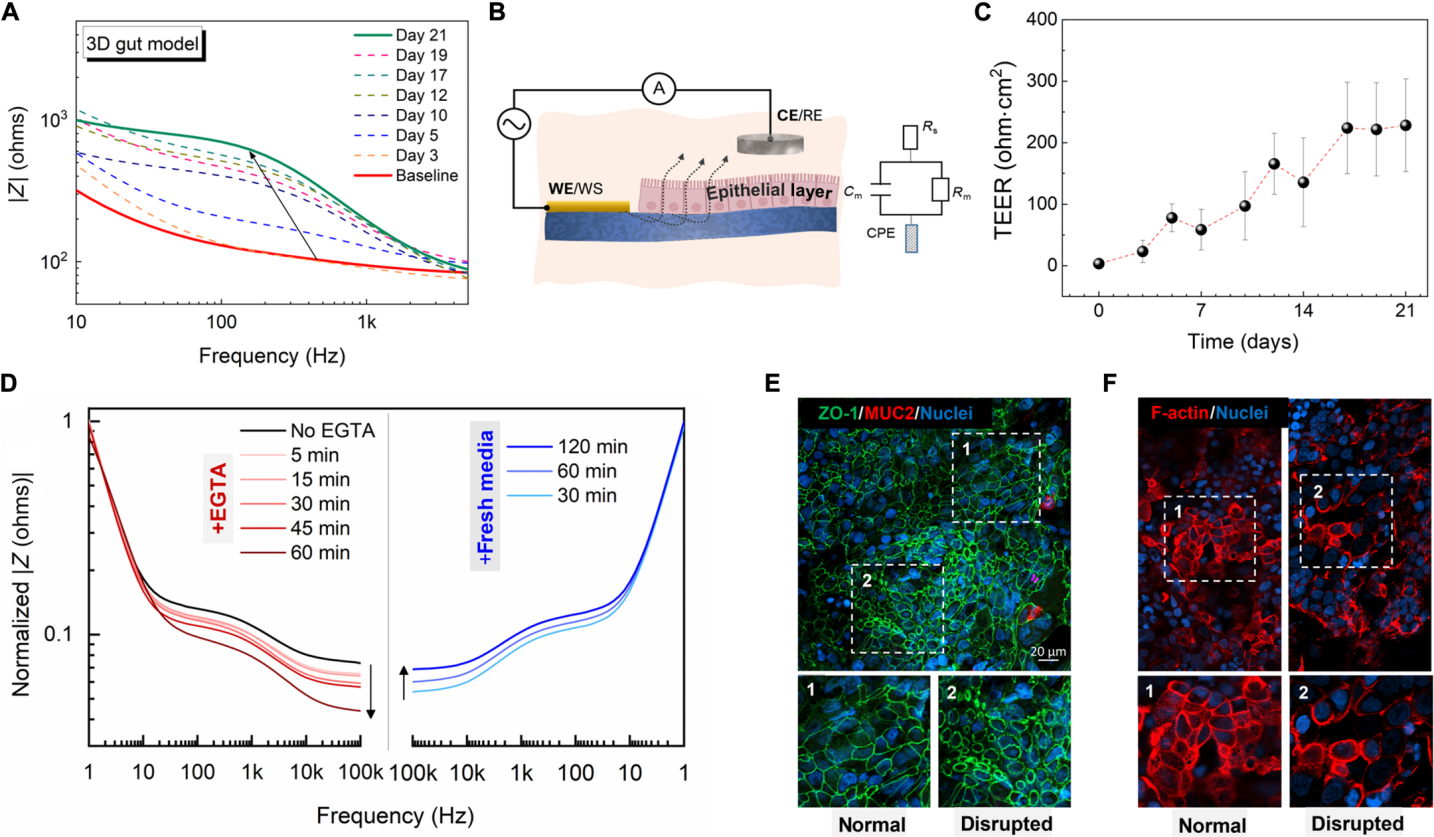
e-transmembrane platform as a tool for culturing and monitoring a 3D model of the human intestine. (**A**) Time-resolved impedance spectra of a 3D gut epithelial model developed in the e-transmembrane device. (**B**) Schematic showing the electrochemical setup of the e-transmembrane and the corresponding equivalent circuit with three elements in series accounting for the electrolyte (*R*_s_), scaffold-membrane (CPE), and the cell layer (*C*_m_//*R*_m_). (**C**) TEER evolution of the epithelial (Caco-2/HT29-MTX) cell monolayer. Data for the Caco-2/HT29-MTX resistance were generated 4 < *N* < 8 different devices and four different experiments. (**D**) Evolution of (Bode) impedance spectra of the gut model upon disruption with EGTA and after its recovery following washing with fresh media. Confocal images of the intestinal epithelium taken after the recovery period of the assay, immunostained for (**E**) ZO-1 tight junction protein (in green), mucin (MUC2 in red), and (**F**) filamentous actin (in red), counterstained for cell nuclei (in blue). The partial recovery of the barrier due to the effects of EGTA is clearly shown in both cases, with domains of the epithelium exhibiting adequate resealing of the junctional space (E1, F1) and other domains where cells are detached from their neighbors, rounded with tight junctions colocalized in their periphery and their filamentous actin relatively disassembled (E2, F2).

We further assess and analyze the capability of the e-transmembrane platform to electrically monitor changes during cell growth in real time. Figure 5A shows the evolution of the impedance magnitude (Bode plots) for the Caco-2/HT29-MTX/fibroblast system cultured in the e-transmembrane device. While the impedance spectra are recorded over a broad frequency domain (0.1 to 10^5^ Hz), for the analysis of the electrical readouts, we neglect the double-layer capacitance-dominated regime (0.1 to 10 Hz), as well as the high frequency range associated with the bulk electrolyte resistance (>10^4^). In accordance with Fig. 4 (E and F), over the first 5 days of culture, fibroblasts exhibited an almost quasi-independent of frequency impedance, with a flat ohmic response at the mid-high frequency regime, characteristic of non–barrier-forming cells. While small variations can be observed between the different fibroblast cultured e-transmembranes, the overall impedance profile remained unchanged. Upon seeding of the IECs, a gradual increase in the impedance magnitude at the range of 10 Hz to 2 kHz was observed, with a plateau region (20 to ~400 Hz) being apparent after the first week of cell culture. This is accompanied by a characteristic angle peak of ~20° to 52°, over the selected time course, as shown in the Bode phase plot in fig. S9. The change in the height of the plateau indicates a change in the resistance of the cell layer, which predominantly reflects the resistance of the paracellular pathway across the cell-cell junctions at this frequency range.

Figure 5C shows the evolution of the TEER value for epithelial cells (Caco-2/HT29-MTX) monitored over the course of ~3 weeks. The extracted TEER values measured here are comparable with published values for Caco-2/HT29-MTX cocultures (although higher here) using commercial impedance analyzers (*25*). The TEER evolution profile shows an increase above baseline levels 3 days after seeding, while it becomes more pronounced at day 12. After this point the resistance progression exhibits a lag phase. The postconfluence stage initiates a second rise in the cellular resistance, due to the undergoing differentiation process, which involves morphological alterations of the epithelial layer (i.e., microvilli and brush border formation) and mucus production, as shown in fig. S4. Note that while fibroblasts and their derived ECM proteins can affect cell attachment, polarization, and junctional properties of the epithelia, no direct contribution has been observed in the extracted cellular resistance of the triculture model.

### Monitoring of epithelial tight junction disruption

To further assess the compatibility of our platform for real-time monitoring of cell tissue and barrier integrity, we test the ability of the e-transmembrane in detecting breaches in the intestinal barrier. Analysis of the integrity of the junctional network of epithelia, such as the intestinal epithelium, which joins adjacent cells together to form physical barriers in vivo, can be indicative of the barrier tissue state, a property that is critical under certain pathophysiological conditions, such as inflammation (*46*). The dependence of tight and adherens junctions on Ca^2+^ is well established in literature (*47*), where depletion of Ca^2+^ results in disruption of the physiological dynamics of the junctional network (i.e., endocytosis of junctional proteins), disaggregation of adherent cells, and breakage of the barrier integrity, reflected in the increased permeability and lower TEER values of the epithelia (*48*). Readdition of Ca^2+^, however, allows for the epithelium to recover its integrity, resealing the intercellular junctional network. This calcium switch method is commonly used to characterize the barrier integrity of epithelia in vitro by exposing the barrier to Ca^2+^ chelators (*49*), such as EGTA (*50*). The apical compartment of the epithelial models in the e-transmembranes were exposed to EGTA, and effects on the barrier integrity are electrically monitored for 60 min, after which fresh, Ca^2+^-containing, complete growth medium was used to wash the cells. Figure 5D shows the evolution of the normalized impedance at different time points (5, 15, 30, 45, and 60 min) during EGTA exposure, as measured by the e-transmembrane platform. As expected, the impedance magnitude is found to decrease over time, losing 29% of its initial value (at 1 kHz after 60 min), while the overall spectra profile remained the same. The EGTA effect is seen to be more pronounced in the mid-high frequency regime, indicative of tight junction disarray and normal function. In addition, the e-transmembrane platform is able to monitor the recovery process after washing out the EGTA-fueled media and replenishing with fresh media. The impedance magnitude is found to be partially recovered, reaching ~95% of the initial value after 120 min in fresh media. Figure 5E illustrates a section of the apical domain at the end of the experiment, revealing the partial recovery of the barrier, with regions of the junctional network exhibiting the physiological morphology (i) and regions where adjacent cells are detached and ZO-1 is internalized and colocalized with individual cell periphery (ii). In addition, we visualized the cell cytoskeleton, by staining for actin filaments, also abundant in the microvilli structures of the apical brush border of intestinal cells. Similar to the ZO-1 immunostaining, two distinct areas in the immunostained sections of the barrier are observed (Fig. 5F); one area exhibits the expected chicken wire–like pattern, with consistent microvilli formation on the apical surface of cells (red dots in the body of cells are the top tip of microvilli), and the other area reveals cell detachment from the layer and perijunctional actin filament disassembly. This is not unexpected since Ca^2+^ is known to be critical for filamentous actin organization of intestinal cells (*51*), associated with the assembly and stability of perijunctional cytoskeleton and, in turn, with the integrity and turnover of epithelial junctions (*52*). Lastly, note that the mucin domains previously observed (see Fig. 4) are not detected here (fig. S10). This can be also attributed to EGTA, which is known to interact with mucin gels, affecting their viscosity and inducing rapid swelling, hydration, and, eventually, dispersion of mucins (*53*). Overall, these findings suggest that the 3D epithelial barrier only partially recovered its integrity upon reintroduction of Ca^2+^ in the extracellular environment. The relatively short recovery period, combined with the high concentration of EGTA used, can explain this partial/incomplete resealing of the barrier. However, the key point of this imaging assay is that it validates the capability of the e-transmembrane device to sense alterations in the barrier integrity of the 3D epithelium.

## DISCUSSION

This work highlights the first example of a bioelectronic well plate platform based on a CP scaffold membrane (namely, e-transmembrane). The use of e-transmembrane serves as an interfacing porous template for facilitating 3D cell cultures and monitoring cell growth in multiculture systems. We here demonstrate that the e-transmembrane device can operate as an electrode (and as a transistor), the pore size, and morphology of which can be tailored to fit specific applications. Culturing fibroblasts in the optimized e-transmembranes resulted in spatially homogeneous cell distribution throughout the bulk of the scaffold, rendering the biostructures suitable to support other cell types. Coculture of fibroblasts and epithelial or endothelial cells within e-transmembranes resulted in fully confluent monolayers formed on top of stromal tissue generated by the fibroblasts, with expected polarization characteristics. As a proof of concept, a representative 3D model of intestinal epithelium is developed and successfully characterized by means of impedance spectroscopy. Not only is our platform capable of measuring temporal changes of barrier tissue formation, but it is also able to sense breaches in the epithelium caused by chemical agents. Our extracted impedance parameters allow easy comparison with traditional 2D barrier tissue measurements for ease of validation. In contrast to other in vitro biochips, e-transmembrane offers a facile approach to generate and simultaneously measure 3D cell cultures, as well as barrier function of organotypic models (i.e., airway epithelium, epidermis, and blood brain barrier), as a more accurate mimic of animal (human) physiology. While conventional rigid (plastic) membranes used in well plates or chips are limited to variations in the pore size and thickness, e-transmembranes can be engineered for purpose via tailoring of their microtopography, (bio)chemistry, porosity, conductivity, and stiffness. Thus, we believe that the e-transmembrane device has the potential to incubate promising developments for 3D in vitro systems with a significant impact on drug screening and therapeutics given its suitability for higher throughput measurements. Future work is concentrated on expanding the repertoire of tissues to lung, blood brain barrier, esophagus, and vaginal epithelia for a host of drug discovery and disease modeling applications.

## MATERIALS AND METHODS

### e-transmembrane preparation

PEDOT:PSS dispersion (Clevios PH-1000, Heraeus) was mixed with 0.75% (w/v) 4-dodecylbenzenesulfonic acid (Sigma-Aldrich) and 3% (v/v) 3-glycidoxypropyltrimethoxysilane (Sigma-Aldrich) and filtered (0.8 μm; Sartorius). The solution was ultrasonicated following each addition for 15 min with the temperature maintained <20°C. The resulting mixture was pipetted into a 48-well plate (Thermo Scientific BioLite, cell-culture treated) at 500 μl per well. The well plate was freeze-dried (VirTis Advantage Plus); the freezing stage swept the temperature of the sample from 5° to −38°C at a rate of 0.25°C/min at 0.5 mbar. In the drying (sublimation) phase, the temperature was swept from −38°C to room temperature over a period of 10 hours at 0.1 mbar. The resulting ice-templated samples were thermally treated on a hotplate at 70°C overnight to allow for cross-linking. The resulting scaffolds were removed from the well plate glued on pre-prepared PDMS supports and subsequently sliced using a microvibratome in the transverse plane (relative to well plate orientation) in PBS (1×) to a thickness between 100 and 600 μm. A speed between 0.06 and 0.08 mm/s and an oscillation amplitude of 0.8 mm yield the most optimum trade-off between time and section quality. Higher values were found to either compress the sample or perform nonuniform sectioning. The slices were trimmed using an 8-mm biopsy punch and stored in deionized water (Milli-Q) at 4°C before use.

### Regulating the pore size and morphology of the e-transmembranes using additives

For the experiments with the cryoprotectants, DMSO (Sigma-Aldrich) or MeOH was mixed with the pristine PEDOT:PSS solution at a concentration of 1 and 2.0% (v/v), respectively. The solution was kept in ice and subsequently stored in 4°C. For the PEDOT:PSS/PVA-based e-transmembranes, 4 weight % (wt%) PVA (Sigma-Aldrich) in H_2_O was mixed with pristine PEDOT:PSS in 1:2 (v/v) ratio. The mixture was stirred thoroughly and kept in 4°C before use. Similarly, maltose and cellobiose (Sigma-Aldrich) were first diluted in water in 2 and 4 wt%, respectively, and then mixed with pristine PEDOT:PSS solution in 1:2 (v/v) ratio. The resulting mixtures were then pipetted (500 μl) inside the wells of a 48-well plate and freeze-dried using the abovementioned conditions. After the freeze-drying process, the samples were annealed overnight on a hotplate at 70°C to promote cross-linking.

### Preparation of devices for cell seeding

After the e-transmembrane platform is fully assembled, it undergoes sterilization. Each device is placed in dedicated wells of a sterile 24-well plate, prefilled with 70% ethanol, while ethanol is also added in the apical compartment so the lid electrodes can be fully immersed in it. The lids are thoroughly sprayed and wiped with ethanol as well. Sterilization is followed by careful and thorough rinsing with sterile water and PBS and then the e-transmembrane scaffolds are immersed in complete growth media of TIF cells (see below) for 2 hours to allow for protein adhesion on the scaffold surface. For this step, each e-transmembrane is removed from the well, the bottom of which is then filled with media. Then, each device is carefully moved back to the well, gently pushed in with a pair of tweezers to remove any air bubbles that could be trapped in the basal compartment. Medium is also added in the apical compartment of the devices, making sure that the reference electrodes/CEs can be fully immersed in each well, to facilitate electrical monitoring (at this stage, we take the first measurements). Before seeding, scaffolds are rinsed with fresh medium and, with a pair of tweezers, placed in the middle of the dedicated well in a nontreated 12-well plate.

### Gelatin phantom electrodes

A positive master mold was 3D printed (Anycubic Photon S, Clear Resin); the mold was postprocessed and coated with a release agent (Electrolube CPL200H) to prevent cure inhibition of the PDMS. PDMS (10:1) was cast into the master mold and cured in a vacuum chamber overnight at ambient temperature and then cross-linked at 65°C for 8 hours. The PDMS negative was removed from the master and cleaned with isopropyl alcohol and ethanol (70%). A second, flat, featureless PDMS film with the same dimensions as the PDMS negative mold was fabricated similarly. Gelatin (porcine skin; G1890, Sigma-Aldrich) powder [10 or 20% (w/v)] was dissolved in KCl aqueous solution (0.1 or 3 M) at room temperature and then mixed with a magnetic stir bar at 60 Hz in a water bath maintained at 35°C for at least 1 hour. Using a syringe, the PDMS negative mold was filled with gelatin solution, and the PDMS film was placed atop the mold, displacing excess gelatin. The filled mold was placed on ice for at least 30 min. Circular gelatin films were extracted from the mold by way of scalpel and wetted spatula. The hydrophobicity of the PDMS makes the use of a release agent unnecessary. The e-transmembrane device was assembled as per usual except for the gelatin film, which was placed on the apical aspect of the e-transmembrane, with the apical gasket placed atop the film. EIS was conducted similarly to the rest of the devices. The protocol is graphically depicted in fig. S11.

### Epithelial cell model

The generation of the 3D intestinal epithelial barrier on the e-transmembranes was based on the biological model and the two-step seeding strategy, originally described here (*17*), adapted to the devices used here. PEDOT:PSS scaffolds (400 μm thick) were used for all the experiments unless otherwise stated. First, a supporting lamina propria layer was established in the bulk of scaffolds by culturing human TIFs labeled with RFP (TIF LifeAct; a gift from E. Van Obberghen-Schilling, Institut de Biologie de Valrose). TIF LifeAct (P9-12) were routinely maintained in Advanced DMEM (Dulbecco’s modified Eagle’s medium; Gibco) supplemented with 20% fetal bovine serum (FBS; Sigma-Aldrich), 1% glutamine (Gibco), 2% HEPES (N-2-hydroxyethylpiperazine-N-2-ethane sulfonic acid) (Gibco), 0.5% penicillin-streptomycin (10,000 U/ml; Gibco), and 0.1% gentamycin (Sigma-Aldrich), harvested with 0.25% trypsin. A total of 250,000 fibroblasts, suspended in 70 μl of medium, were “dropped” on the top surface of each scaffold disc with a pipette. Cells were then incubated (within the nontreated well plate) for 2 hours to allow for cell attachment and adhesion. After 2 hours, each device was carefully moved back to its dedicated well of a sterile (treated/cell culture grade) 24-well plate, prefilled with fresh cell growth media. Again, with a pair of tweezers, each device was gently squeezed in the well, displacing the air out while pushing in, to avoid formation and trapping of bubbles in the basal compartment. Fresh cell growth medium was also added in the apical compartment, making sure that the CEs/reference electrodes of the lid would be fully immersed in each device. These TIF 3D cultures were maintained for 4 days to allow cells to infiltrate the scaffolds and secrete ECM proteins. Medium was changed once.

On day 5 of TIF culture, the IECs were seeded in the devices to generate the epithelial barrier model. To this end, the 3:1 Caco-2 and HT29-MTX coculture described here was used (referred to as IEC coculture). Both Caco-2 and HT29-MTX cells were purchased from ECACC and were routinely maintained before all experiments. Caco-2 cells (P50-58) were cultured in Advanced DMEM (Gibco), supplemented with 10% FBS (Sigma-Aldrich), 1% M Glutamine (Glutamax-1; Invitrogen), 1% penicillin-streptomycin (10,000 U/ml; Gibco), and 0.1% gentamycin (Sigma-Aldrich). HT29-MTX-E12 (P54-59) cells were maintained in Advanced DMEM (Gibco), supplemented with 10% FBS (Sigma-Aldrich) and 1% penicillin-streptomycin (10,000 U/ml; Gibco). Before seeding, cells were harvested with 0.5 and 0.25% trypsin and mixed to a single-cell suspension in the desired ratio. Each preseeded with TIF device was removed from the 24-well plate, after removing the cell medium from both compartments, and placed in the center of a dedicated well in a sterile, nontreated 12-well plate. Again, 250,000 IEC cells (3:1 Caco-2:HT29-MTX), suspended in 70 μl of medium, were dropped on top of each scaffold with a pipette, and cells were incubated for 2 hours to allow for cell attachment and adhesion, before moving them back in the e-transmembrane platform, with the same method as described before, filled with fresh coculture medium, the composition of which was tweaked as described here. This triculture system was maintained for another 3 weeks (total culture period, 26 to 27 days), with media being changed every other day, before samples were fixed with 4% paraformaldehyde (PFA; Thermo Fisher Scientific) and stored in PBS for optical characterization.

### Endothelial cell seeding

TIFs were seeded onto the e-transmembrane scaffolds. Scaffolds were suspended in 1 ml of TIF media (see below) and placed onto an orbital shaker (IKA KS 260 Basic, IKA; Staufen, Germany) rotating at 60 rpm. A total of 200,000 TIFs in 75 μl of medium were pipetted into the well containing the scaffold in the device and left on the orbital shaker. After 24 hours, the medium was removed and replaced with 2 ml of fresh TIF medium. The devices were cultured under static conditions for the following 3 days. HUVECs (Lonza Biosciences, Basel, Switzerland) were added into the system in the same manner, applying 50,000 HUVECs within 75 μl of HUVEC medium (EGM-2; Lonza Biosciences, Basel, Switzerland). After 24 hours, the medium was removed and replaced with fresh HUVEC medium. These samples were left to culture statically for 2 days, at which point the orbital shaker was reinitiated to simulate blood flow. Cultures were carried out for an additional 7 days before samples were fixed for imaging.

### Calcium switch assay

To investigate the potential of the 3D e-transmembranes to detect disruption of the epithelial barrier in real time, we used a calcium switch assay. More specifically, we added 500 mM ethylene glycol tetraacetic acid in the apical compartment of the gut epithelium 3D e-transmembranes, diluted from stock in cell growth medium (Alfa Aesar EGTA, 0.5 M aqueous solution; J60767) and monitored its effects for 60 min. Cells were then washed three times with fresh complete growth medium, containing Ca^2+^. The recovery of the epithelial barrier was also monitored electrically for the following 2 hours before cells were fixed with 4% PFA, washed with PBS, and stored for imaging.

### Electrochemical characterization

Impedance measurements were performed using a potentiostat (Metrohm Autolab) equipped with a frequency response analysis module. As shown in the device geometry in Fig. 1, a two-electrode electrochemical cell was used for the EIS measurements of the e-transmembranes. Pt or stainless steel mesh of 20 mm by 20 mm was folded several times and used as a CE. The CE was fixed at the lid and immersed inside the top compartment of the well. The distance between CE and WE remained the same for all the measurements. The applied AC voltage was 0.01 V, and measurements were carried out at 0-V DC potential versus open circuit potential. EIS measurements were performed from 100 kHz to 100 mHz. Simulation and fitting of the curves were carried out using EC-Lab (Bio-Logic) software. The model described in fig. S8 was used for the generation of the *R* and *C* parameters. For the calculation of the pseudo-capacitance associated with a CPE (*Q*), the following formula was used

$$\frac{1}{2\pi {\left(\mathrm{RQ}\right)}^{\frac{1}{a}}}=\frac{1}{2\pi \mathrm{RC}}$$

where *Q* is the magnitude of the CPE and *a* (0 < *a* < 1) is exponent of the CPE.

TEER values were extracted by using the following equation: TEER = *R*_cell_ × *A*, with *R*_cell_ being the net resistance of the cell layer calculated using the EIS data and *A* being the surface of the electrode. In our case, *A* is equal to *A* = π*r*^2^, with *r* being the radius of the circular membrane (*r* ~ 3.6 mm).

Transistor characteristics were taken using a Keithley 2612B source meter and customized LabVIEW software. The two Au O-ring electrodes were assigned as source-drain, while the gate electrode was the Ag/AgCl. The electrical measurements were performed in PBS.

### Immunofluorescence

All samples were fixed in 4% PFA (Thermo Fisher Scientific) for 20 min at room temperature. After thorough washing with PBS, each scaffold was stored at 4°C until it is ready to use for optical analysis. Before immunostaining, cells were permeabilized in 0.1% (v/v) Triton X-100 (Fisher) for 15 min and then blocked for nonspecific binding with 1% (w/v) bovine serum albumin (Fisher) and 0.1% (v/v) Tween 20 (Fisher) in PBS for 1 hour at room temperature. To label the samples for actin filaments, tight junction protein ZO-1, MUC2, VE-cadherin, and nuclei, the following primary and secondary antibodies were used: Rb polyclonal anti–ZO-1 (617300, Thermo Fisher Scientific), Ms monoclonal anti-MUC2 (ab11197, Abcam), bisbenzimide H (Hoechst 33342; ab228511, Abcam), goat-anti-rabbit Alexa Fluor 488 (ab150077, Abcam), goat–anti-mouse Alexa Fluor 647 (ab150115, Abcam), Phalloidin-iFluor 594 Reagent (ab176757, Abcam), and Rb monoclonal VE-cadherin (D87F2, Cell Signaling Technology). Slices were then placed on microscopy plates and were maintained hydrated with PBS during imaging with the epifluorescence/confocal microscope Axio Observer Z1 LSM 800 (ZEISS).

### Scanning electron microscopy

SEM imaging was used to characterize the morphological properties of both uncultured and cultured e-transmembranes (Leo Variable pressure SEM, ZEIS GmbH). For the cultured samples, the cells in the scaffold were first fixed in 4% PFA for 20 min at room temperature. Then, they were washed thoroughly in PBS, dehydrated in a graded ethanol series, and soaked in hexamethyldisilazane (HMDS; Sigma-Aldrich) solution until complete dehydration (overnight in pure HMDS). Last, before proceeding with the imaging process, the treated samples were mounted on a conductive adhesion tape.

## Notes

View/request a protocol for this paper from *Bio-protocol*.
