# Supplementary material for: Organic electronic transmembrane device for hosting and monitoring 3D cell cultures
Source: Sci Adv. 2022 Sep 16;8(37):eabo4761. doi: 10.1126/sciadv.abo4761 (PMC9481123; doi:10.1126/sciadv.abo4761)
Supplement: Supplementary file 1 — Figs. S1 to S11 Table S1 [file sciadv.abo4761_sm.pdf]

Science Advances
AAAS

Supplementary Materials for

Organic electronic transmembrane device for hosting and monitoring 3D cell cultures

Charalampos Pitsalidis *et al.*

Corresponding author: Róisín M. Owens, rmo37@cam.ac.uk;
Charalampos Pitsalidis, charalampos.pitsalidis@ku.ac.ae

Sci. Adv. **8**, eabo4761 (2022)
DOI: 10.1126/sciadv.abo4761

This PDF file includes:

Figs. S1 to S11
Table S1

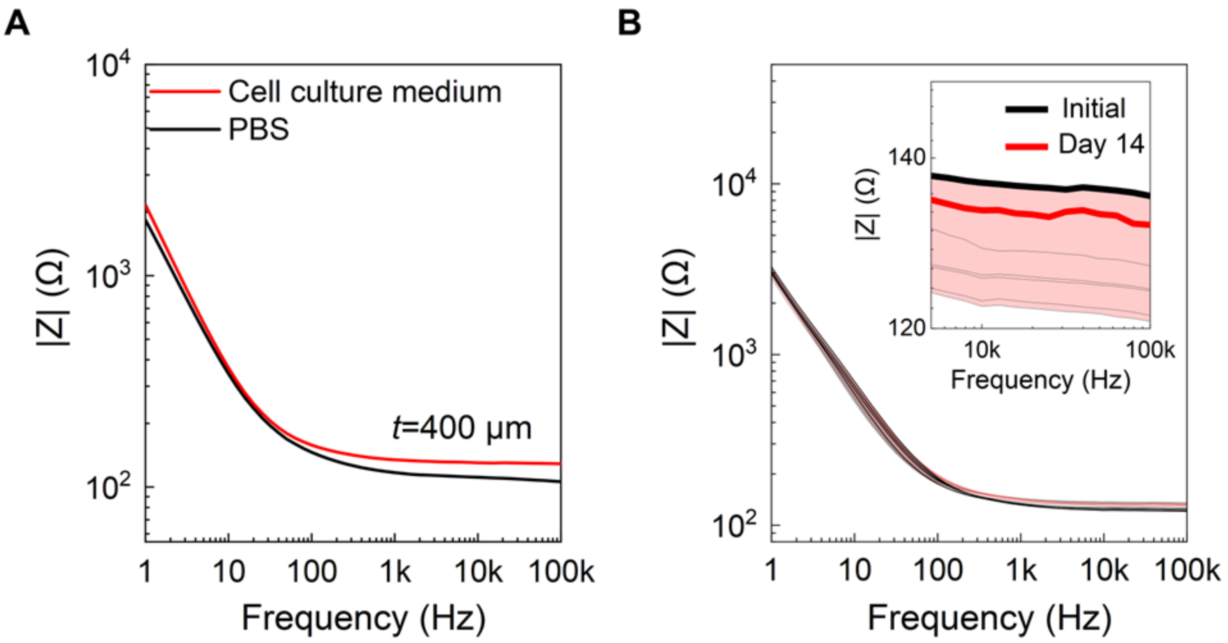

FIG. S1. Long-term stability of PEDOT:PSS e-transmembrane devices in cell culture medium and comparative plots for operation in PBS and cell culture medium. **(A)** Comparative EIS measurements of PEDOT:PSS e-transmembranes operating in PBS and cell culture medium. **(B)** EIS spectra showing variations in the electrochemical performance of an e-transmembrane device for different timepoints over a period of 14 days in cell culture media. The thickness of the e-transmembrane is 400 µm.

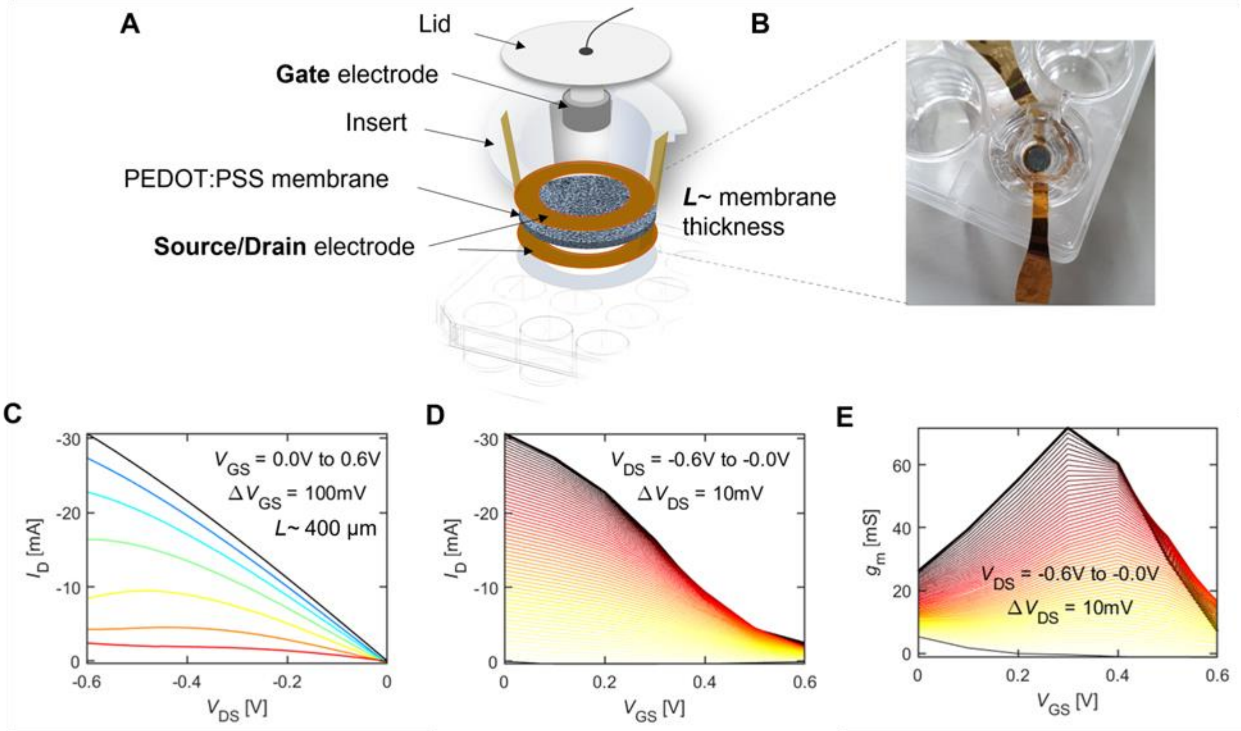

FIG. S2. OECT based on e-transmembranes and device operation. (A) OECT device architecture based on e-transmembrane setup and **(B)** a corresponding photograph of the actual device. e-transmembrane is sandwiched between two flexible (Au) O-ring electrodes forming the source and drain of the transistor. The thickness of the e-transmembrane (400 μm) defines the channel length of the transistor. (**C**) Output and **(D)** transfer OECT characteristics measured using the e-transmembrane OECTs and the corresponding (**E**) transconductance (g_m) plots as a function of gate voltage (V_{GS}).

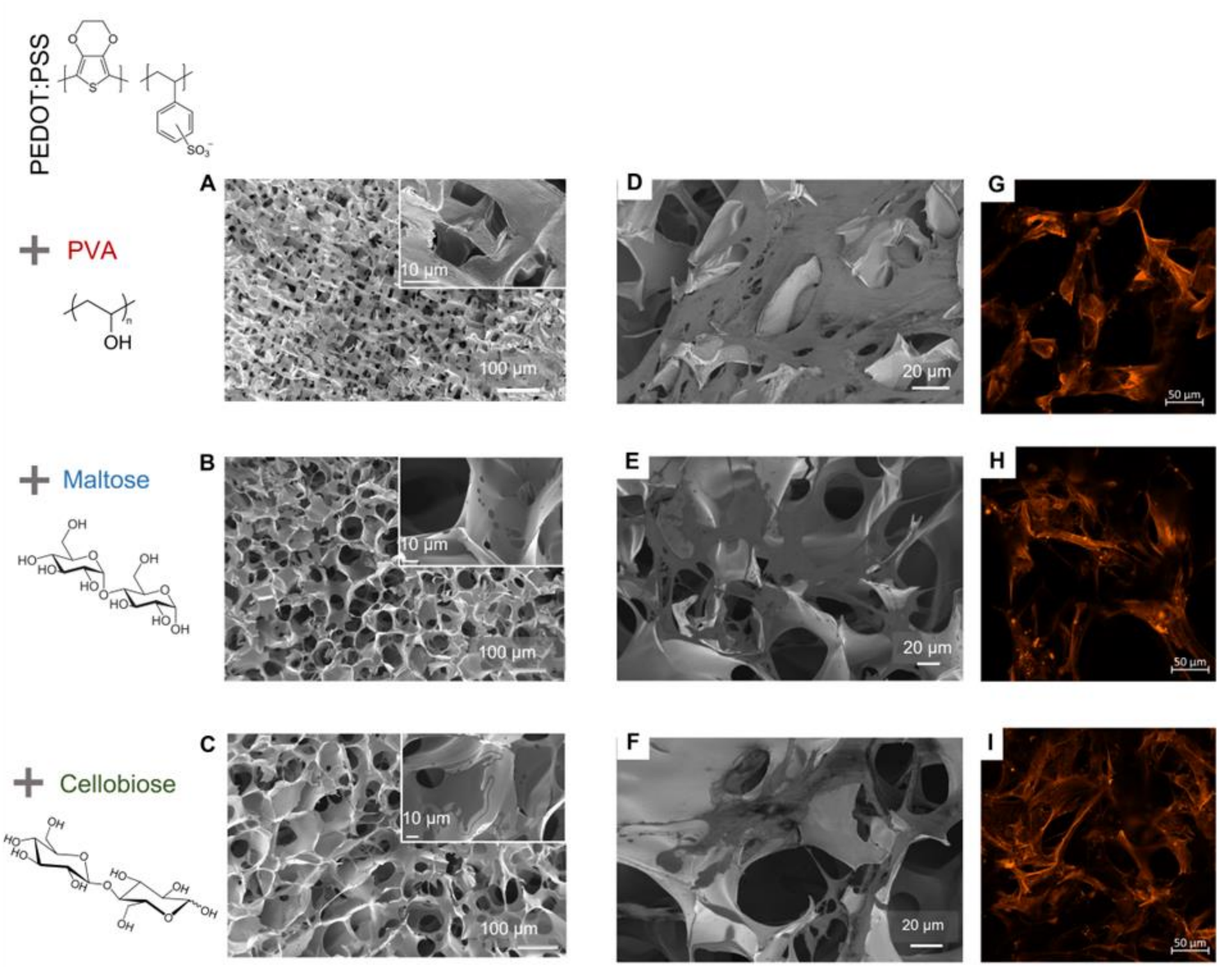

FIG. S3 Morphology and cytocompatibility experiments of e-transmembrane composites. e-transmembranes based on PEDOT:PSS composites. SEM images showing the porous morphology of (**A**) PEDOT:PSS/PVA, (**B**) PEDOT:PSS/Maltose and (**C**) PEDOT:PSS /Cellobiose e-transmembranes. Inserts are high magnification SEM images showing the pore surface and structure. Corresponding (**D-F**) SEM and (**G-I**) immunofluorescence images of cultured e-transmembranes with telomerase immortalized fibroblasts (TIF).

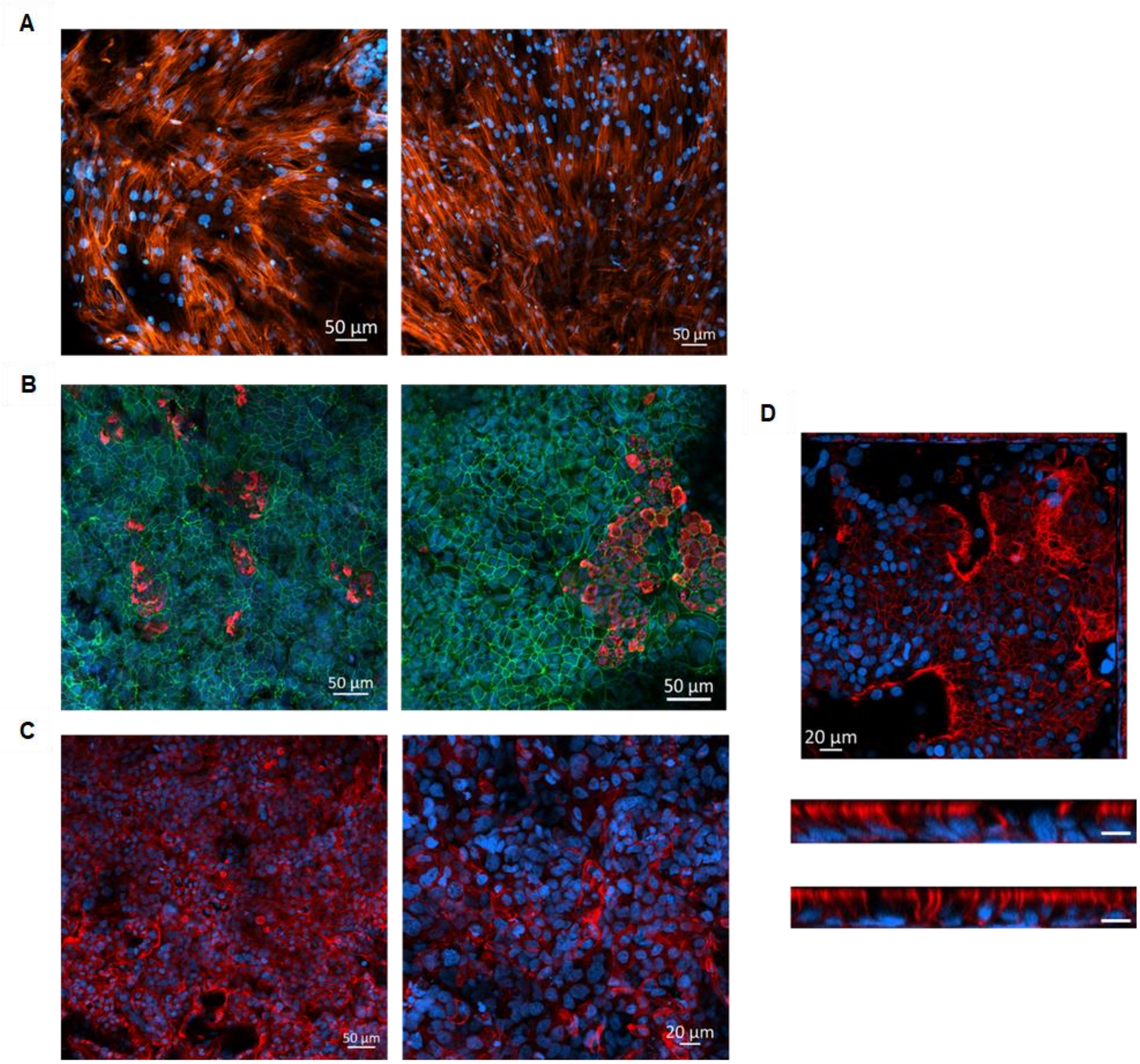

FIG. S4. Confocal image microscopy of fibroblasts and intestinal epithelial layer. Confocal microscopy images **(A)** of fibroblasts (TIFs with actin cytoskeleton labelled for Red Fluorescent Protein), demonstrating cell infiltration and formation of the characteristic fibrillar network in the bulk of the e-transmembrane scaffolds at the end of the ~1 month experiment, **(B)** of immunostained intestinal epithelial layer on the apical domain of the e-transmembranes, illustrating the typical chicken-like pattern of ZO-1 protein distribution (in green) in the tight junction network, overlaid by MUC2 (in red) and **(C)** the filamentous actin of the cytoskeleton (in red), and **(D)** of the apical brush border of the cell layer and its microvilli filamentous actin (in red). The bottom panels of (D) are magnified y/z (top) and x/z (bottom) orthogonal views of the top panel, offering a closer look at the polarisation of the intestinal layer and its characteristic microvilli, rich in actin filaments (in red; scale bars 10 µm). In all cases cells were counterstained for nuclei (in blue).

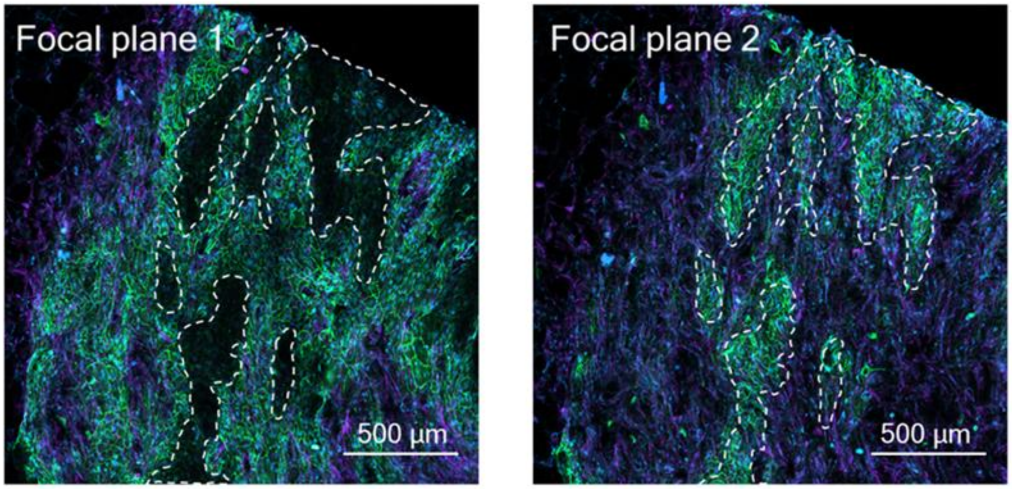

FIG. S5. Low magnification confocal microscopy images of an immunostained vascular endothelial cell layer on top of fibroblasts on the e-transmembrane as measured from different focal planes. Low magnification confocal microscopy images of an immunostained vascular endothelial cell layer (endothelial junctions VE-cadherin tagged with green), on top of fibroblasts (tagged with purple) on the e-transmembrane as measured from different focal planes.

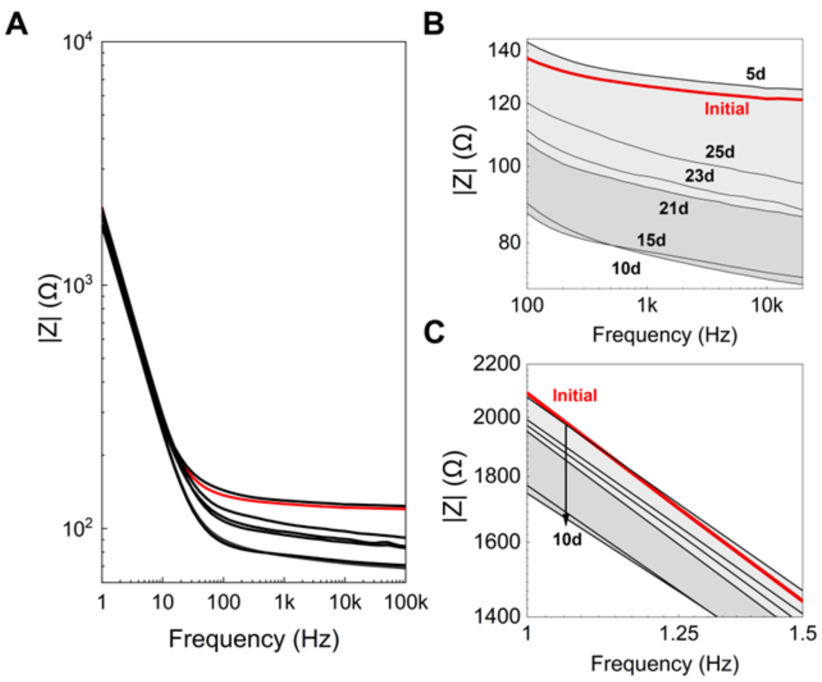

FIG. S6. EIS evolution spectra of fibroblast cell culture over 25 days. (**A**) EIS evolution spectra of TIF cell culture over a period of 25 days. Red line corresponds to measurements taken from uncultured e-transmembranes and black lines corresponds to the TIF cultured e-transmembranes at different timepoints. EIS spectra showing the evolution at the (B) high and (C) low frequency regimes.

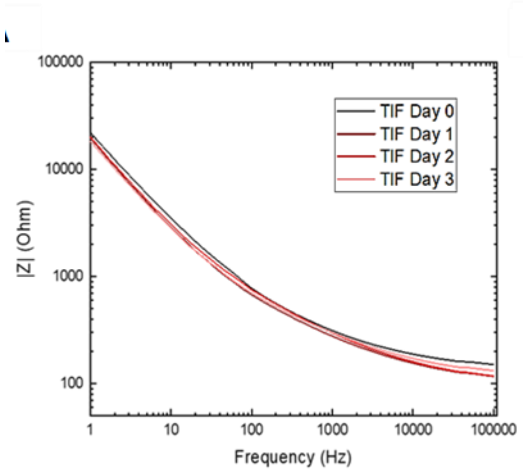

FIG. S7. **Impedance spectra of fibroblasts cultured on Transwell filter membrane.** Impedance (Bode plots) spectra of TIFs cultured on Transwell filter membranes, showing the evolution of the impedance magnitude over 4 days in culture impedance (N=3).

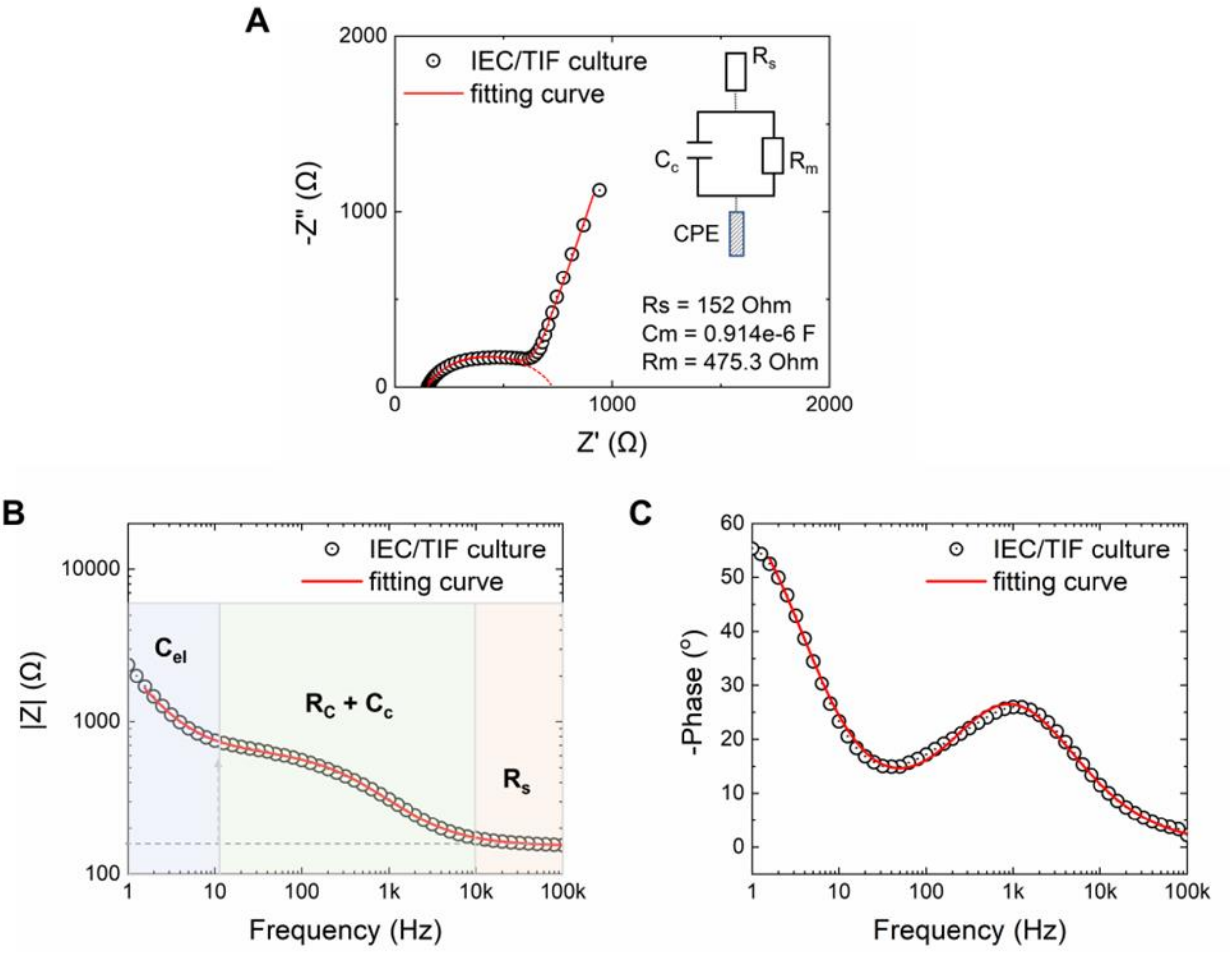

FIG. S8. **Description and fitting of the analysis of EIS spectra.** Fitting EIS data generated by (TIF/Caco-2/HT29-MTX) cultured e-transmembrane, exhibiting typical cell barrier-forming characteristics. (**A**) Nyquist plot and the corresponding equivalent circuit. Red line represents the fit of the curve. The semicircle which corresponds to the associated cell barrier can be fitted using an R-parallel-CPE (constant phase element) for non-ideal capacitors. (**B**) Fit of the Bode magnitude plot and the corresponding (**C**) phase plot.

Table S1. Measured fit parameters from the Caco-2/HT29-MT/TIF and HUVEC/TIF models of FIG. 4. The number of devices is N=3.

	Resistance R_C (Ω)			Mean R_C	Capacitance C_c (µF)			Mean C_c
**Caco-2/HT29-MTX/TIF**	661.9	453.3	467.7	**527.6 ±116.5**	0.81	0.33	0.69	**0.60 ±0.26**
**HUVEC/TIF**	174.4	281	277.5	**244.3 ±60.6**	0.14	0.33	0.43	**0.30 ±0.15**

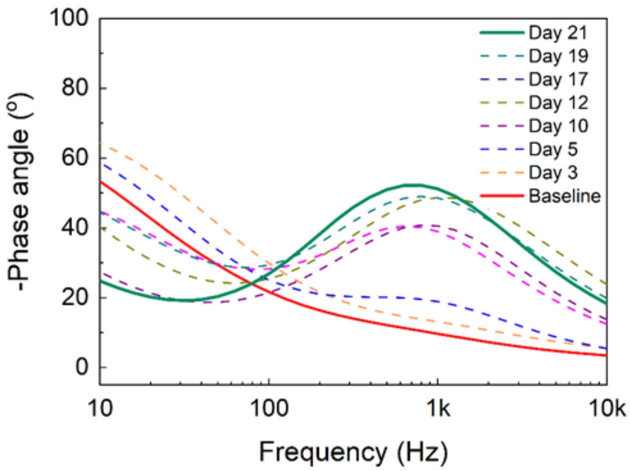

FIG. S9. **Representative Bode phase evolution curves at different timepoints for a 3D intestinal model.** Representative Bode phase evolution curves at different timepoints for a 3D intestinal model developed in a e-transmembrane device. Baseline curve represents day 5 for fibroblasts (TIF) and day 0 for intestinal epithelial cells.

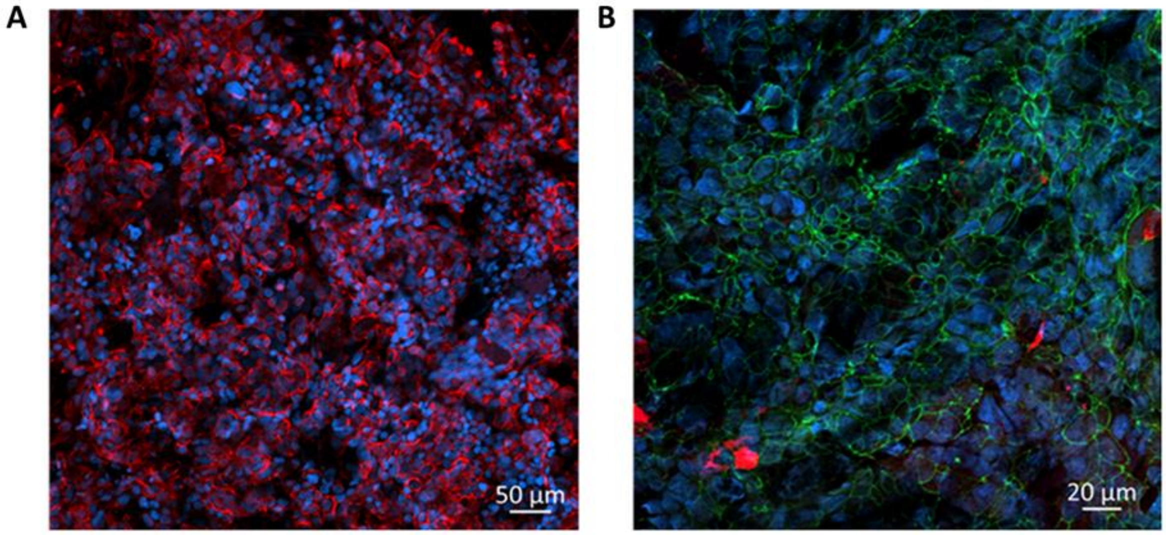

FIG. S10. Confocal images of the intestinal epithelium after the recovery period of the Ca^{2+} switch assay. Confocal images (additional to **Figure 5 E, F**) of the intestinal epithelium after the recovery period of the Ca^{2+} switch assay, showing partial recovery of the barrier. **(A)** The image illustrates unaffected/recovered domains of the layer and domainss that exhibit signs of barrier disruption, with rounded cells and disassembled filamentous actin (in red). **(B)** The image shows the intestinal layer immunostained for ZO-1 tight junction protein (in green) and mucin (MUC2 in red), revealing domains of the tight junction network adequately resealed and other domains where ZO-1 is co-localised in the periphery of cells that are rounded and detached from the adjacent cells. In both panels, cells are counterstained for nuclei (in blue).

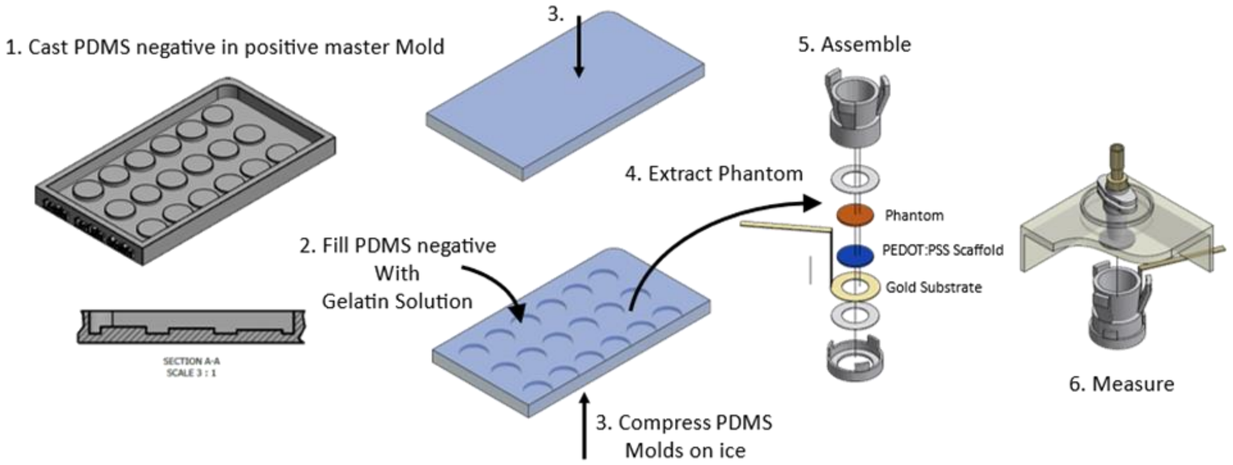

FIG. S11: **Flow diagram illustrating the protocol for generating and assembling phantoms for the e-transmembrane device.** A positive master mold is 3D printer using a resin DLP printer – the mold allows for three different thicknesses of gelatin film, all with the same diameter. The mold is coated with a release agent which does not induce cure inhibition in the PDMS (Electrolube CPL). PDMS (10:1) is cast into the positive master mold and cured overnight under vacuum. The negative PDMS mold is extracted and the gelatin solution is drop cast into the depressions. A second PDMS slap is placed atop the negative mold, flush with the mold surface, and the loose assembly is placed on ice for 20 minutes. The gelatin films are extracted using wetted tweezers and spatulas and inserted into the e-transmembrane assembly such that the film covers the apical aspect of the PEDOT:PSS scaffold.
